# Immunomodulatory Potential of the Polysaccharide-Rich Extract from Edible Cyanobacterium *Nostoc commune*

**DOI:** 10.3390/medsci3040112

**Published:** 2015-11-04

**Authors:** Hui-Fen Liao, Tai-Jung Wu, Jia-Liang Tai, Meng-Chun Chi, Long-Liu Lin

**Affiliations:** 1 Department of Biochemical Science and Technology, National Chiayi University, 300 Syuefu Road, Chiayi City 60004, Taiwan; E-Mail: s1000312@mail.ncyu.edu.tw; 2 Department of Applied Chemistry, National Chiayi University, 300 Syuefu Road, Chiayi City 60004, Taiwan; E-Mail: s0910324@mail.ncyu.edu.tw; 3 Sung Yu Technology Co., Ltd., 7 Dali Road, Rende District, Tainan City 71754, Taiwan; E-Mail: tai@sung-yu.com.tw

**Keywords:** *Nostoc commune*, polysaccharide-rich extract, immunomoduration, macrophages, leukemia

## Abstract

A dry sample of *Nostoc commune* from an organic farm in Pingtung city (Taiwan) was used to prepare polysaccharide-rich (NCPS) extract. The conditioned medium (CM) from NCPS-treated human peripheral blood (PB)-mononuclear cells (MNC) effectively inhibited the growth of human leukemic U937 cells and triggered differentiation of U937 monoblast cells into monocytic/macrophagic lines. Cytokine levels in MNC-CMs showed upregulation of granulocyte/macrophage-colony stimulatory factor and IL-1β and downregulation of IL-6 and IL-17 upon treatment with NCPS. Moreover, murine macrophage RAW264.7 cells treated with NCPS exhibited the stimulatory effects of nitric oxide and superoxide secretion, indicating that NCPS might activate the immunity of macrophages. Collectively, the present study demonstrates that NCPS from *N. commune* could be potentially used for macrophage activation and consequently inhibited the leukemic cell growth and induced monocytic/macrophagic differentiation.

## 1. Introduction

Cyanobacterium *Nostoc commune* is a conspicuous component of microbial populations in *terrestrial* environments, especially those associated with nutrient-poor soils and limestones [1,2,3]. The geographical distribution of *N. commune* ranges from polar to tropical regions. Its biomes cover deserts, semi-deserts, dry grasslands, and rock surfaces [4,5,6,7]. This microbe can survive long periods of frost and drought as inactive crusts, but the gelatinous colony rapidly swells and reactivates respiration and photosynthesis when water becomes available [8,9]. As a primary pioneer organism that can retain water, build up organic material, and assimilate atmospheric nitrogen, *N. commune* facilitates the colonization of other species in extreme habitats and stimulates their growth by improving water, nutrient, and organic matter supply to the ecosystem [3,4,10].

*Nostoc* species have been used as a food delicacy or ingredient of Chinese medicine since the Eastern Jin Dynasty (317–420 AD), as recorded in Compendium of Materia Medica [11]. The known edible *Nostoc* spp. include *N. commune* var *flagelliforme*, *N. parmelioides* (edule), *N*. *ellipsosporum*, *N. verrucosum*, and *N. pruniforme* [12]. Unfortunately, the growth of some species on sports turf and buildings is undesirable [13], and contributes to production of unpleasant odors in drinking water [14]. However, some phenolic extracts from *Nostoc* species are known as human pathogen inhibitors [15], and may be valuable medicinally.

Few studies have revealed that *N. commune* contains cryptophycin, a compound with inhibition potential of cancer cell growth [16], antiviral agents [17,18], anti-infective metabolites [19], and an extracellular diterpenoid with antibacterial activity [20]. A cholesterol-lowering effect of *N. commune* was also reported in rats fed with a high cholesterol diet and this effect has been attributed to its high fiber content [21,22]. Due to the pharmacological and nutraceutical value of *N. commune*, this microbe has received increasing attention, and the market demand has grown drastically during the last decade. Nevertheless, research works on the biological activities of polysaccharide-rich extract from this microbe are still limited, especially investigations on immunomodulatory potential of NCPS. In the present study, effects of the polysaccharide-rich extract on inducing the differentiation of U937 monoblast cells into monocytes/macrophages, activation of superoxide and nitric oxide production, and phagocytosis of yeast by murine macrophage RAW264.7 cells were investigated.

## 2. Materials and Methods

### 2.1. Materials, Chemicals, and Cell Lines

Dry sample of *N. commune* was obtained in March 2012 from an organic farm in Laubei Village, Neipu Township (22°63′ N and 120°60′ E; Pingtung City, Taiwan). Unless indicated otherwise, all other chemicals and solvents were of analytical grade and purchased from Wako Pure Chemicals Industries, Ltd. (Osaka, Japan), Sigma-Aldrich Fine Chemicals (St. Louis, MO, USA), and Merck (Darmstadt, Germany).

Human monoblastoid leukemia U937 cells (ATCC^®^ CRL1593.2™) were cultured in Roswell Park Memorial Institute (RPMI) 1640 medium (Gibco, Grand Island, NY, USA) supplemented with 10% heat-inactivated fetal bovine serum (FBS; Hyclone Laboratories, Inc., South Logan, UT, USA) and maintained in a state of exponential growth for assaying the effect of polysaccharide-rich extract from *N. commune* (NCPS) on cell viability and differentiation. Dulbecco’s Modified Eagle Medium (DMEM) containing 10% heat-sterilized FBS was used to cultivate murine macrophage RAW264.7 cells (ATCC^®^ TIB-71™).

### 2.2. Preparation of Polysaccharide-Rich Extract

Rehydrated sample of *N. commune* was thoroughly washed in tap water and rinsed once with distilled water. The washed samples were then dried for overnight in an oven at 50 °C and pulverized to a fine powder using a Starlite blender (Model SL-999). To prepare NCPS, the pulverized powder (20 g) was suspended in 600 mL of distilled water at room temperature for overnight. After that, the crude extract was recovered by centrifugation at 12,000× *g* for 10 min and concentrated to approximately 300 mL by a rotary evaporator. Ethanol (~99%) was subsequently added to the crude extract at a ratio of 7 to 3. The resultant precipitate (~1.7 g) was collected by centrifugation and washed twice sequentially with ethanol and acetone. After being fully dissolved in distilled water, the suspension was filtered, lyophilized and stored at 4 °C until used.

To test the sensitivity of NCPS towards heat and proteinase K treatment, the lyophilized sample (1 g) was re-suspended in 10 mL of phosphate buffer saline (PBS). Portions (each 5 mL) of the suspension were individually subjected to heat treatment at 95 °C for 3 h [23], and proteinase K (50 μg/mL, Sigma-Aldrich, Inc., Saint Louis, MI, USA) treatment at 37 °C for 2 h. Following proteinase K treatment, the enzyme was inactivated by the addition of phenylmethylsulfonyl fluoride.

### 2.3. Cell Viability Assay

Peripheral blood samples were acquired from untreated healthy donors (20–30 years old) with the approval of Institutional Review Board and the legal requirements of informed consent. Mononuclear cells (MNCs) from human blood were separated by centrifugation on a Ficoll-Hypaque solution with a density of 1.077 g/mL (Pharmacia Fine Chemicals, Pistakaway, NJ, USA). The harvested MNCs at a concentration of 2 × 10^6^ cells/mL were cultivated in an RPMI 1640 medium containing 10% heat-inactivated FBS and 0.5–100 mg/mL of NCPS at 37 °C in a humidified 5% CO_2_ incubator for 24 h. The cell-free supernatants were then collected as the conditioned medium MNC-CM, and stored at −70 °C until used. During the experiments, phytohemagglutinin (PHA; Difco, MI, USA) was used as the positive control. To rule out the possible contamination of endotoxin, the NCPS-stimulated cells were prepared in the presence of 50 μg/mL polymyxin B (Sigma-Aldrich) in cell viability and differentiation experiments.

For cell viability assay, RAW264.7 cells at an initial concentration of 1 × 10^5^ cells/mL were individually incubated in 35 mm Petri-dishes in the presence of various concentrations of NCPS for two days, while the same concentration of U937 cells was co-incubated with 30% (*v*/*v*) MNC-CM for three days. The number of viable cells in a cell suspension was counted using the trypan blue dye exclusion test [24].

### 2.4. Superoxide Production Assay

The generation of cytoplasmic superoxide by differentiated myeloid cells was monitored with the nitroblue tetrazolium (NBT) reduction assay [25]. Firstly, the NCPS-treated RAW264.7 and NCPS-MNC-CM-co-cultivated U937 cells were collected, suspended in the respective medium at a final concentration of 1 × 10^6^ cells/mL, and incubated with an equal volume of NBT test stock solution containing 2 mg NBT and 1 μM phorbol myristate acetate per mL of DMSO at 37 °C for 30 min.

### 2.5. Assay for Nitric Oxide (NO) Production

RAW264.7 macrophage cells were treated with NCPS as described above and the concentration of NO released from the cells was determined by Greiss reagent (Sigma-Aldrich) using the standard curve of NO_2_^−^. The assay mixtures contained 100 μL of culture supernatant and an equal volume of Greiss reagent, and the thoroughly mixed samples were left at ambient temperature for 10 min before the absorbance measurement at a wavelength of 540 nm. To eliminate the interaction between sample and Greiss reagent, NO concentration of the culture medium without cells was simultaneously measured to calibrate that obtained with the cells.

### 2.6. Cytokine Profiles

The cytokine levels expressed in MNC-CM were carried out according to procedures recommended in the Proteome Profiler^TM^ Array manual (R&D systems, Inc., Minneapolis, MN, USA). The expression profile of 36 different cytokines in MNC-CMs was exposed using enhanced chemiluminescence (ECL) (Amersham Pharmacia 211 Biotech, Aylesbury, UK) assay. Relative expression levels were determined by densitometry using ImageJ software (Version 1.36b, NIH, Bethesda, MD, USA).

### 2.7. Assay for Phagocytosis

The phagocytic activity was determined as described previously [26]. Briefly, the yeast stock suspension at a concentration of 1 × 10^8^ cells/mL was made by mixing *Saccharomyces cerevisiae* cells with PBS. The cells were collected, washed, re-suspended in FBS-containing RPMI1640 medium, and incubated in the yeast suspension (adjust to approximately 4 × 10^6^ cells/mL) with NCPS-treated RAW264.7 cells at 37 °C for 2 h. Thereafter, the yeast cells were placed on a glass slide and observed under an inverted microscope. The percentage of yeast cells with a nucleus was calculated from 200 independent cells.

### 2.8. Statistical Analysis

The experimental results were expressed as the mean ± standard deviation (SD) of three independent trials. Statistical comparisons were carried out on the basis of two-tailed Student’s *t*-test or analysis of variance. A value of *p* < 0.05 is used to mark the statistically significant difference. All statistical analyses were performed using SigmaStat software (Jandel Scientific, San Jose, CA, USA).

## 3. Results and Discussion

### 3.1. Effects of MNC-CMs on Leukemia U937 Cells

**Figure 1:**
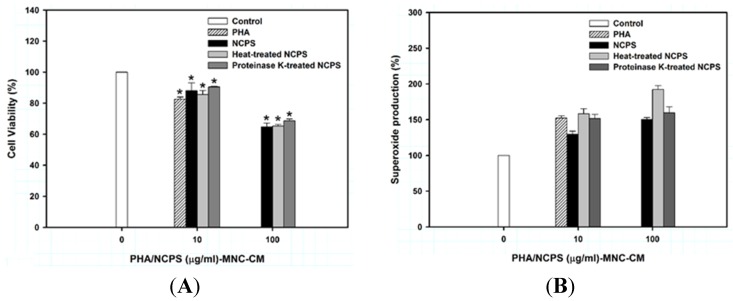
Cell viability (**A**) and superoxide production (**B**) of U937 cells upon treatment with MNC-CMs for three days. PHA was used as the positive control. * indicates that there is a statistically significant difference between control and treatment groups (*p* < 0.05).

Earlier, an experimental model of anti-leukemic immunity had been developed to assess the immunomudulatory activity of natural polysaccharides in which the polysaccharide-stimulated MNCs produce many different types of cytokines to inhibit the growth and differentiation of human leukemia U937 cells [26,27]. In this study, the CM collected from NCPS-treated MNCs showed a significant inhibitory effect on the viability of human leukemia U937 cells (Figure 1A). As a positive control, PHA-treated MNC-CM had similar inhibitory activity at the respective concentrations. Both heat and proteinase K treatments did not abolish the inhibitory effect (Figure 1A), suggesting that the protein portion is not a key component to the biological activity of the polysaccharide-rich extract. Structural characterization of the released heteroglycans from *N. commune* and other *Nostoc* spp. has shown that they are consisting of a similar 1-4-linked xylogalactoglucan backbone with d-ribofuranose and 3-*O*-[(*R*)-1-carboxyethyl]-d-glucuronic acid (nosturonic acid) pendant groups [1,23]. Desiccated crusts of *N. commune* are brittle and friable but have the consistency of cartilage when rehydrated. The massive and rapid swelling of desiccated colonies following rainfall is sufficiently striking that it was even the subject of medieval folklore [28]. These rheological properties of the extracellular heteroglycan, as well as its resistance to degradation *in situ*, its ability to prevent membrane fusion upon removal of water, and its significant contribution to the dry weight of colonies emphasize the pivotal role for this biopolymer in the biology of *N. commune*, especially the capacity for desiccation tolerance. Beyond its physiological role, this type of biopolymer may be responsible for the immunomudulatory activity of NCPS.

Production of cytoplasmic superoxide, which can be monitored by the ability to reduce NBT, is a typical characteristic of mature monocytes/macrophages [29]. Treatment of U937 cells with NCPS-MNC-CM, heat-treated NCPS-MNC-CM, and proteinase K-treated NCPS-MNC-CM resulted in a marked increase in the percentage (up to 50.1% ± 5.2%) of superoxide-producing cells (Figure 1B). Superoxide production by the treated cells occurred in a dose-independent manner. However, neither NCPS alone nor normal MNC-CM displayed this effect.

**Figure 2:**
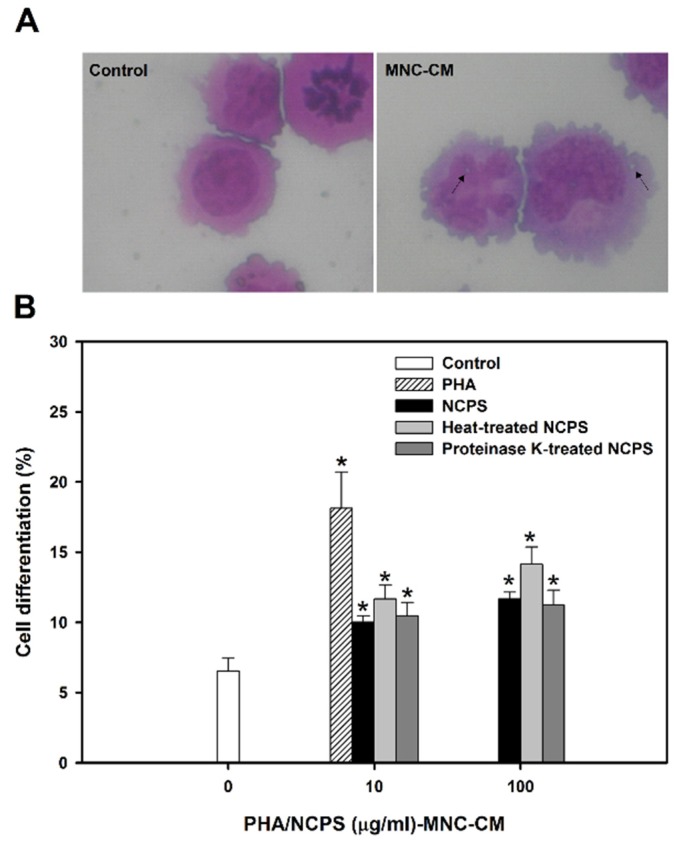
Cell morphology (**A**) and monocytic/macrophagic differentiation (**B**) of U937 cells upon treatment with MNC-CMs for three days. PHA was used as the positive control. * indicates that there is a statistically significant difference between control and treatment groups (*p* < 0.05).

As shown in Figure 2A, the prepared MNC-CMs significantly triggered differentiation of U937 cells into mature monocytes. The morphology of monocytes includes the increase of cytosol/nuclear ratio, expression of vacuoles in cytosol and formation of pseudopods in cell membrane. The percentage of mature monocytes was significantly increased from 6.54% ± 0.91% (untreated control) to 10.03% ± 0.44%, 11.68% ± 0.99% and 10.47% ± 0.96% by MNC-CM prepared with NCPS, heat-treated NCPS and proteinase K-treated NCPS, respectively (Figure 2B). The experimental results clearly indicate that NCPS-MNC-CM can stimulate U937 cells toward monocytic differentiation. Moreover, the stimulation seemed to be a dose-independent event due to there being no significant difference in the percentage of mature monocytes between concentrations 10 and 100 μg/mL.

There are two possible routes for a natural compound to act on leukemic cells, either by the direct inhibition of tumor cell growth or by the physiologic stimulation of immunocompetent cells to secrete differentiation-inducing factors and cytokines [30]. In this study, NCPS did not have significant inhibition on leukemic U937 cells (data not shown), but treatment of U937 cells with NCPS-MNC-CM resulted in marked effects, including inhibition of cell growth and stimulation of monocytic differentiation.

It is noteworthy that we extracted the water-soluble fraction and then collected the polysaccharide-rich portion (NCPS) by ethanol precipitation [31]. The parallel bioactivity assay of NCPS by heat treatment and proteinase K proteo-hydrolysis demonstrated that the character of NCPS is heat-stable and the major active component is likely to be polysaccharide. To gain more insight into the immunomodulatory activity of NCPS, our ongoing work will focus on the structural characterization of complex heteroglycans from *N. commune* by gel filtration, FPLC, GC-MS, and NMR.

### 3.2. Cytokine Levels of Various MNC-CMs

**Figure 3:**
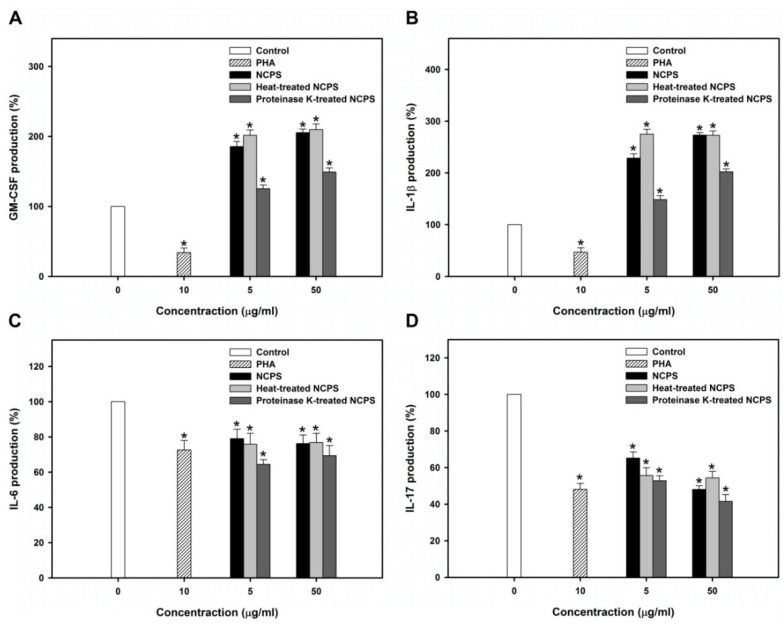
Levels of GM-CSF (**A**), IL-1β (**B**), IL-6 (**C**), and IL-17 (**D**) in MNC-CMs that were produced from MNC with the treatment of PHA, NCPS, heat-treated NCPS, and proteinase K-treated NCPS. Star indicates that there is a statistically significant difference between control and treatment groups (*p* < 0.05).

MNC contains several types of immune cells, such as monocytes, macrophages, lymphocytes, and natural killer cells [32]. Among the immune cells, various cytokines could be secreted to regulate downstream immune responses and form an immunocompetent network [33]. As shown in Figure S1, the expression profile of 36 different cytokines in MNC-CMs was assayed by a cytokine array kit (Proteome Profiler™ Array, R&D systems). NCPS treatment changed certain cytokine levels in MNC-CMs, including the upregulation of granulocyte/macrophage-colony stimulatory factor (GM-CSF) and interleukin (IL)-1β and downregulation of IL-6 and IL-17 (Figure 3).

GM-CSF is produced from monocytes, macrophages and T lymphocytes and stimulates the survival, proliferation, differentiation, and functional activation of monocytes, granulocytes, and hematopoietic stem cells [34]. The present study showed that NCPS and heat-treated NCPS significantly stimulated MNC to produce GM-CSF more than two-fold. However, proteinase K-treated NCPS only increased GM-CSF production by 25%–50%. These observations suggest that proteins contained in NCPS may have a stimulatory effect on GM-CSF secretion.

IL-1β is produced from activated macrophages and regulates inflammation, apoptosis, differentiation and proliferation of several immune cells [35]. It had been reported that IL-1β inhibits cell growth and induces differentiation in leukemic cells [36]. In this study, NCPS and heat-treated NCPS significantly stimulated MNC to produce IL-1β by more than 1.7-fold. Proteinase K-treated NCPS also exhibited a 40% to 98% enhancement in IL-1β production. These results further indicate that the protein portion of NCPS has a significantly stimulatory effect on IL-1β secretion.

IL-6 and IL-17 are two important cytokines that induce inflammatory response [37]. This study demonstrated that NCPS decreased IL-6 and IL-17 levels to about 75% and 50% at the concentration of 50 μg/mL, suggesting that NCPS might reduce the inflammatory effect. IL-6 has been reported to be overexpressed in several inflammatory diseases, for example: rheumatoid arthritis, osteoporosis, psoriasis, and cancers [38]. Like IL-6, IL-17 also has high correlation with inflammatory and autoimmune diseases [39]. Elimination of IL-6 and IL-17 levels has therefore become a strategy for inflammatory disease treatment [40,41].

Overall, the results indicated that NCPS may not induce inflammatory response, but effective for stimulating MNC to secrete cytokines, such as GM-CSF and IL-1β. Cytokines contained in NCPS-MNC-CM also have potential to inhibit cell growth and induce monocytic differentiation of leukemic U937 cells.

### 3.3. Stimulatory Effect of NCPS on Macrophage RAW264.7 Cells

**Figure 4:**
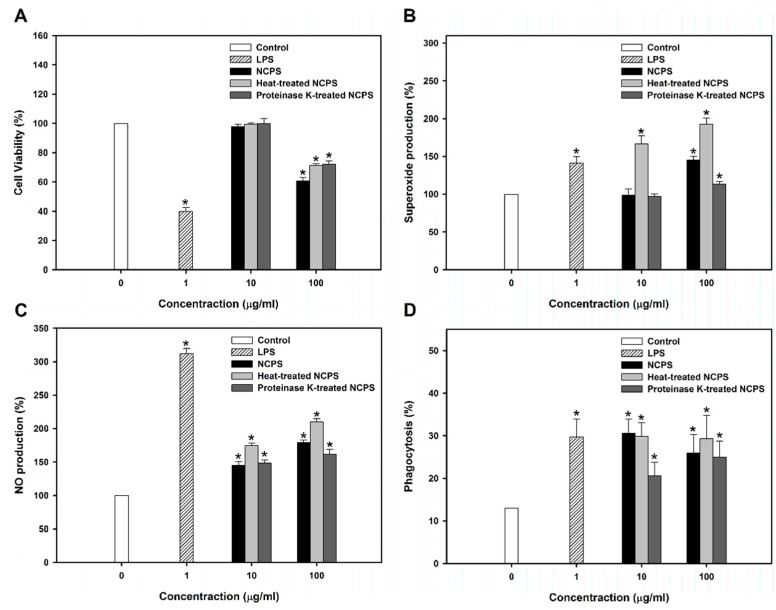
Cell viability (**A**), superoxide production (**B**), nitric oxide synthesis (**C**), and phagocytosis (**D**) of RAW264.7 cells upon treatment with NCPS for two days. * indicates that there is a statistically significant difference between control and treatment groups (*p* < 0.05).

For further investigation of NCPS on macrophage activation, murine RAW264.7 cells were used. Figure 4A shows that NCPS at low concentration (10 μg/mL) did not change cell viability. High concentration of NCPS (100 μg/mL) decreased cell viability by about 30%. Upon immune response initiation, NO and superoxide produced by the activated macrophages can kill the pathogens and the recognition of infection leads to induction of adaptive immunity through activation of antigen-presenting cells [42]. The present study demonstrated that NCPS effectively stimulated the production of superoxide and NO in RAW264.7 cells (Figure 4B,C). The NCPS-treated RAW264.7 cells increased the effect of phagocytosis of yeast (Figure 4D and Figure S2), indicating that NCPS has the ability to activate macrophages.

To rule out possible endotoxin contamination, 50 μg/mL polymyxin B was added in the experiments of NO production assay (Figure S3). The results showed that LPS was blocked by polymyxin B, while NCPS did not change the effect, suggesting non-existent or insignificant endotoxin contamination in NCPS.

## 4. Conclusions and Future Perspectives

In summary, we demonstrated that the polysaccharide-rich fraction (NCPS)-treated MNC-CM effectively inhibited the growth of human leukemic U937 cells and triggered the differentiation of U937 monoblast cells into monocytic/macrophagic lines. Cytokine levels in NCPS-MNC-CM showed the upregulation of GM-CSF and IL-1β and downregulation of IL-6 and IL-17. Moreover, NCPS may activate the immunity of macrophage RAW264.7 cells. Therefore, NCPS from this cyanobacterium has potential for macrophage activation and consequently anti-leukemic effects. *N. commune* is a prominent species of microbial populations worldwide, distributed from the tropics to the polar regions of the earth. Traditionally, some species of *Nostoc*, including *N. commune*, have been used as a food source or as medicine to treat illness. The present results showed that the ethanolic extract from the edible cyanobacterium had potent immunomodulatory activity. Thus, the natural compounds derived from the extract might be useful to improve human health. Further investigations on the development of *N. commune* for functional food supplements would benefit from its immunomodulatory activity.

## Supplementary Materials

Supplementary File 1

## References

[1] Helm R.F., Huang Z., Edwards D., Leeson H., Peery W., Potts M. (2000). Structural characterization of the released polysaccharide of desiccation-tolerant *Nostoc commune* DRH-1. J. Bacteriol..

[2] Shirkey B., Kovarcik D.P., Wright D.J., Wilmoth G., Prickett T.F., Gregory E.M., Potts M. (2000). Active Fe-containing superoxide dismutase and abundant *sodF* mRNA in *Nostoc commune* (Cyanobacteria) after years of desiccation. J. Bacteriol..

[3] Novis P.M., Whitebread D., Gregorich E.G., Hunt J.E., Sparrow A.D., Hopkin D.W., Elberling B., Greenfield L.G. (2007). Annual carbon fixation in terrestrial populations of *Nostoc commune* (Cyanobacterium) from an Antarctic dry valley is driven by temperature regime. Glob. Chang. Biol..

[4] Holst J., Butterbach-Bahl K., Liy C.Y., Cheng X.H., Kaiser A.J., Schnitzler J.P., Zechmeister-Boltenstern S., Bryggemann N. (2009). Dinitrogen fixation by biological soil crusts in an Inner Mongolian steppe. Biol. Fertil. Soils.

[5] Marsh J., Nouvet S., Sanborn P., Coxson D. (2006). Composition and function of biological soil crust communities along topographic gradients in grasslands of central interior British Columbia (Chilcotin) and southwestern Yukon (Kluane). Can. J. Biol..

[6] Aboal M., Cristobal C.J., Marin-Murcia J.P. (2010). About the presence of *N. commune* var *flagelliforme* (Nostocaceae, Cyanophyceae) on clay soils from arid regions of south east Spain. Acta Bot. Malacit..

[7] Ramirez M., Hernandez-Marine M., Mateo P., Berrendeo E., Roldan M. (2011). Polyplastic approach and adaptive strategies of *Nostoc*
*cf. commune* (Nostocales, Nostocaceae) growing on Mayan monuments. Fottea.

[8] Scherer S., Ernst A., Chen T.W., Böger P. (1984). Rewetting of drought resistant blue-green algae: Time course of water uptake and reappearance of respiration, photosynthesis, and nitrogen fixation. Oecologia.

[9] Satoh K., Hirai M., Nishio J., Yamaji T., Kashino Y., Koike H. (2002). Recovery of photosynthetic systems during rewetting is quite rapid in a terrestrial cyanobacterium, *Nostoc commune*. Plant Cell Physiol..

[10] Sand-Jensen K., Baastrup-Spohr L., Winkel A., Møller C.L., Borum J., Brodersen K., Lindell T., Staehr P.A. (2010). Plant distribution patterns and adaptation in a limestone quarry on Øland. Sven. Bot. Tidsskr..

[11] Li S.Z. (1977). Compendium of Materia Medica.

[12] Chu H.J., Tsang C.T. (1988). Research and utilization of cyanobacteria in China: A report. Archiv Hydrobiol..

[13] Baldwin N.A., Whitton B.A. (1992). Cyanobacteria and eukaryotic algae occurring in sports turf and amenity grasslands: A review. J. Appl. Phycol..

[14] Wnorowski A.U. (1992). Tastes and odours in the aquatic environment: A review. Water SA.

[15] De Cano M.S., de Mulé M.C.Z., de Caire G.Z., de Halperin D.R. (1990). Inhibition of *Candida albicans* and *Staphylococcus aureus* by phenolic compounds from the terrestrial cyanobacterium *Nostoc muscorum*. J. Appl. Phycol..

[16] Smith C.D., Zhang X., Mooberry S.L., Patterson G.M., Moore R.E. (1994). Cryptophycin: A new antimicrotuble agent active against drug-resistant cells. Cancer Res..

[17] Knubel G., Larsen L.K., Moore R.E., Levine I.A., Patterson G.M. (1990). Cytotoxic, antiviral indolocarbazoles from a blue-green alga belonging to the Nostocacae. J. Antibiot. Tokyo.

[18] Esser M.T., Mori T., Mondor I., Sattentau Q.J., Dey B., Berger E.A., Boyd M.R., Lifson J.D. (1999). Cyanovirin-*N* binds to gp120 to interfere with CD4-dependent human immunodeficiency virus type I virion binding, fusion, and infectivity but does not affect the CD4 binding site on gp120 or soluble CD4-induced conformational changes in gp120. J. Virol..

[19] Broniatowska B., Allmendinger A., Kaiser M., Montamat-Sicotte D., Hingley-Wilson S., Lalvani A., Guiry M., Blunden G., Tasdemir D. (2011). Antiprotozoal, antitubercular and cytotoxic potential of cyanobacterial (blue-green algal) extracts from Ireland. Nat. Prod. Commun..

[20] Jaki B., Orjala J., Sticher O. (1999). A novel extracellular diterpenoid with antibacterial activity from the cyanobacterium *Nostoc commune*. J. Nat. Prod..

[21] Hori K., Ishibashi G., Okita T. (1994). Hypocholesterolemic effect of blue-green alga, ishikurage (*Nostoc commune*), in rats fed atherogenic diet. Plant Foods Hum. Nutr..

[22] Rasmussen H.E., Blobaum K.R., Jesch E.D., Ku C.S., Park Y.K., Lu F., Carr T.P., Lee J.Y. (2009). Hypocholesterolemic effect of *Nostoc commune* var. *sphaeroides Kützing*, an edible blue-green alga. Eur. J. Nutr..

[23] Jensen S., Petersen B.O., Omarsdottir S., Paulsen B.S., Duus J.Ø., Olafsdottir E.S. (2013). Structural characterization of a complex heteroglycan from the cyanobacterium *Nostoc commune*. Carbohydr. Polym..

[24] Strober W. (2001). Trypan blue exclusion test of cell viability. Curr. Protoc. Immunol..

[25] Baehner R.L., Nathan D.G. (1968). Quantitative nitroblue tetrazolium test in chronic granulomatous disease. N. Engl. J. Med..

[26] Liao H.F., Chen Y.Y., Yang Y.C., Wang C.S., Chen Y.J. (2006). Rice (*Oryza sativa* L.) inhibits growth and induces differentiation of human leukemic U937 cells through activation of peripheral blood mononuclear cells. Food Chem. Toxicol..

[27] Liao H.F., Chou C.J., Wu S.H., Khoo K.H., Chen C.F., Wang S.Y. (2001). Isolation and characterization of an active compound from black soybean (*Glycine max* L. Merr.) and its effect on proliferation and differentiation of human leukemic U937 cells. Anticancer Drug.

[28] Potts M. (1997). Etymology of the genus name *Nostoc* (Cyanobacterial). Int. J. Syst. Bacteriol..

[29] Yagisawa M., You A., Yonemaru M., Imajoh-ohmi S., Kanegasaki S., Yazaki Y., Takaku F. (1996). Superoxide release and NADPH oxidase components in mature human phagocytes: Correlation between functional capacity and amount of functional proteins. Biochem. Biophys. Res. Commun..

[30] Ganguly C., Das S. (1994). Plant lectins as inhibitors of tumor growth and modulators of host immune response. Chemotherapy.

[31] Xu J., Guan J., Chen X.J., Zhao J., Li S.P. (2011). Comparison of polysaccharides from different *Dendrobium* using saccharide mapping. J. Pharm. Biomed. Anal..

[32] Zhang M., Huang B. (2012). The multi-differentiation potential of peripheral blood mononuclear cells. Stem Cell Res. Ther..

[33] Liles W.C., van Voorhis W.C. (1995). Nomenclature and biologic significance of cytokines involved in inflammation and the host immune response. J. Infect. Dis..

[34] Armitage J.O. (1998). Emerging applications of recombinant human granulocyte-macrophage colony-stimulating factor. Blood.

[35] Smirnova M.G., Kiselev S.L., Gnuchev N.V., Birchall J.P., Pearson J.P. (2002). Role of the pro-inflammatory cytokines tumor necrosis factor-alpha, interleukin-1beta, interleukin-6 and interleukin-8 in the pathogenesis of the otitis media with effusion. Eur. Cytokine Netw..

[36] Onozaki K., Tamatani T., Hashimoto T., Matsushima K. (1987). Growth inhibition and augmentation of mouse myeloid leukemic cell line differentiation by interleukin 1. Cancer Res..

[37] Wang M.H., Ronsin C., Gesnel M.C., Coupey L., Skeel A., Leonard E.J., Breathnach R. (1994). Identification of the *ron* gene product as the receptor for the human macrophage stimulating protein. Science.

[38] Ishihara K., Hirano T. (2002). IL-6 in autoimmune disease and chronic inflammatory proliferative disease. Cytokine Growth Factor Rev..

[39] Aggarwal S., Gurney A.L. (2002). IL-17: Prototype member of an emerging cytokine family. J. Leukoc. Biol..

[40] Smolen J.S., Maini R.N. (2006). Interleukin-6: A new therapeutic target. Arthritis Res. Ther..

[41] Swardfager W., Winer D.A., Herrmann N., Winer S., Lanctôt K.L. (2013). Interleukin-17 in post-stroke neurodegeneration. Neurosci. Biobehav. Rev..

[42] Jutras I., Desjardins M. (2005). Phagocytosis: At the crossroads of innate and adaptive immunity. Ann. Rev. Cell Dev. Biol..

