# Supplementary material for: Immunomodulatory Potential of the Polysaccharide-Rich Extract from Edible Cyanobacterium Nostoc commune
Source: Med Sci (Basel). 2015 Nov 4;3(4):112–23. doi: 10.3390/medsci3040112 (PMC5635763; doi:10.3390/medsci3040112)
Supplement: Supplementary File 1 [file medsci-03-00112-s001.docx]

Supplementary Materials

| 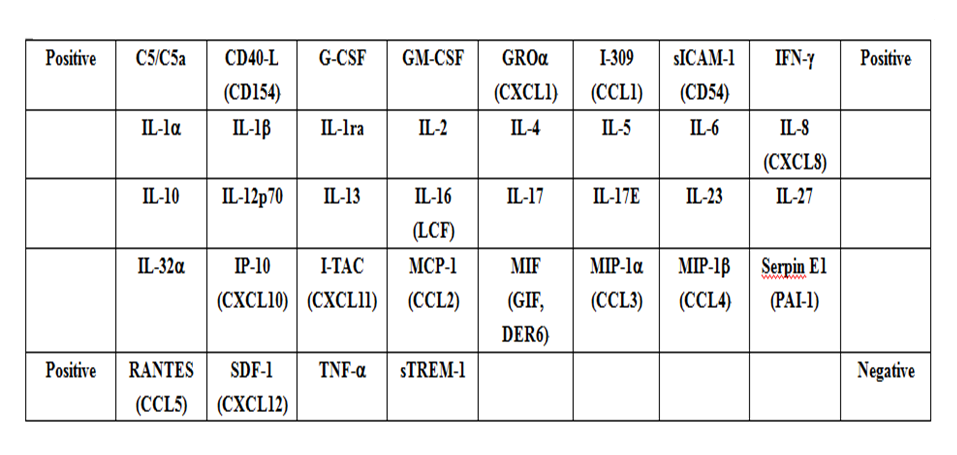 |
| --- |
| (A) |
| 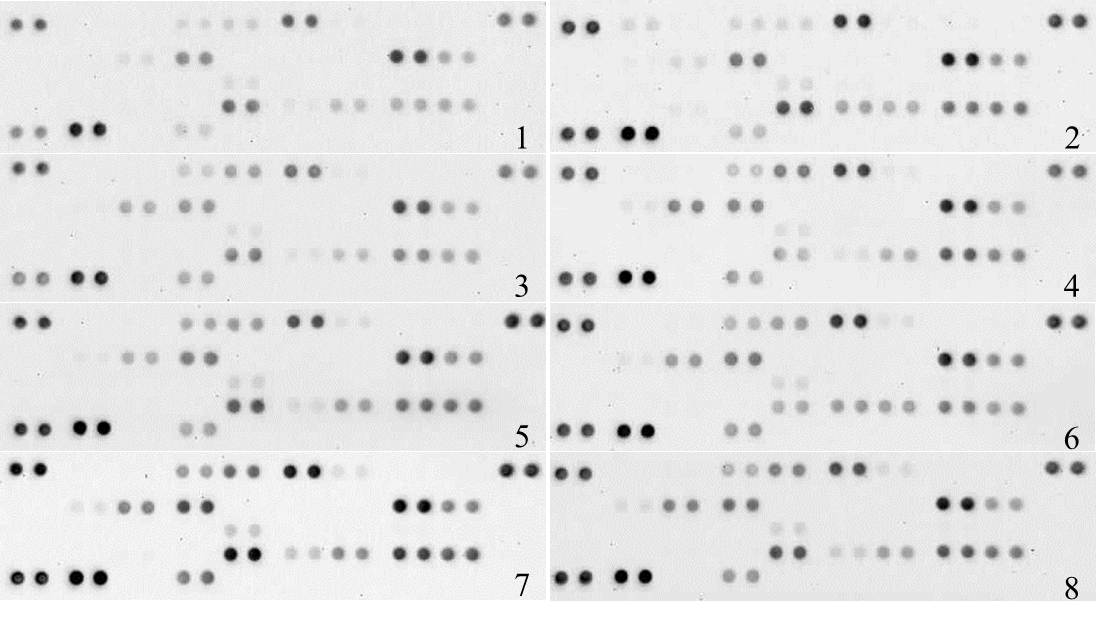 |
| (B) |

**Figure S1.** Cytokine array analysis. Panels: (**A**) A map giving the location of the cytokine antibodies spotted onto the protein chip; (**B**) cytokine levels of MNC-CM with the treatment of panels: (**1**) control; (**2**) 10 μg/mL PHA; (**3**) 5 μg/mL NCPS; (**4**) 50 μg/mL NCPS;
(**5**) 5 μg/mL heat-treated NCPS; (**6**) 50 μg/mL heat-treated NCPS; (**7**) 5 μg/mL proteinase k-treated NCPS; (**8**) 50 μg/mL proteinase k-treated NCPS.

| 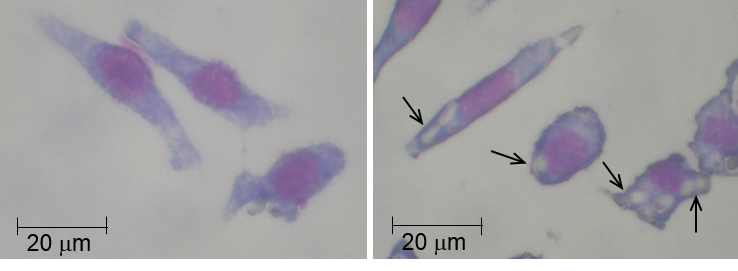 | 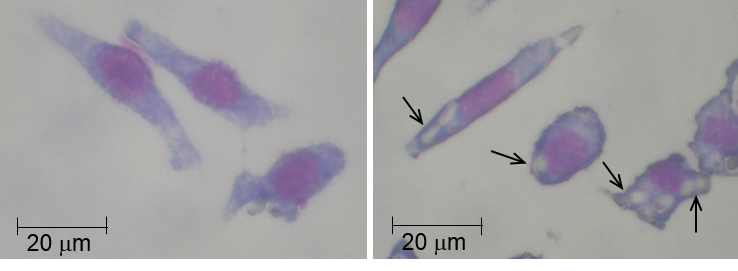 |
| --- | --- |
| (**A**) | (**B**) |

**Figure S2.** Phagocytosis of yeast by RAW264.7 cells. Panels: (**A**) Before treatment;
(**B**) After treatment. The cells were photographed under a fluorescence microscope at a magnification of 1000× with a Nikon Optiphod transmitted light microscope
(Nikon Instech Co., Kanagawa, Japan). The internalization of yeast by the cells was indicated by an arrow.


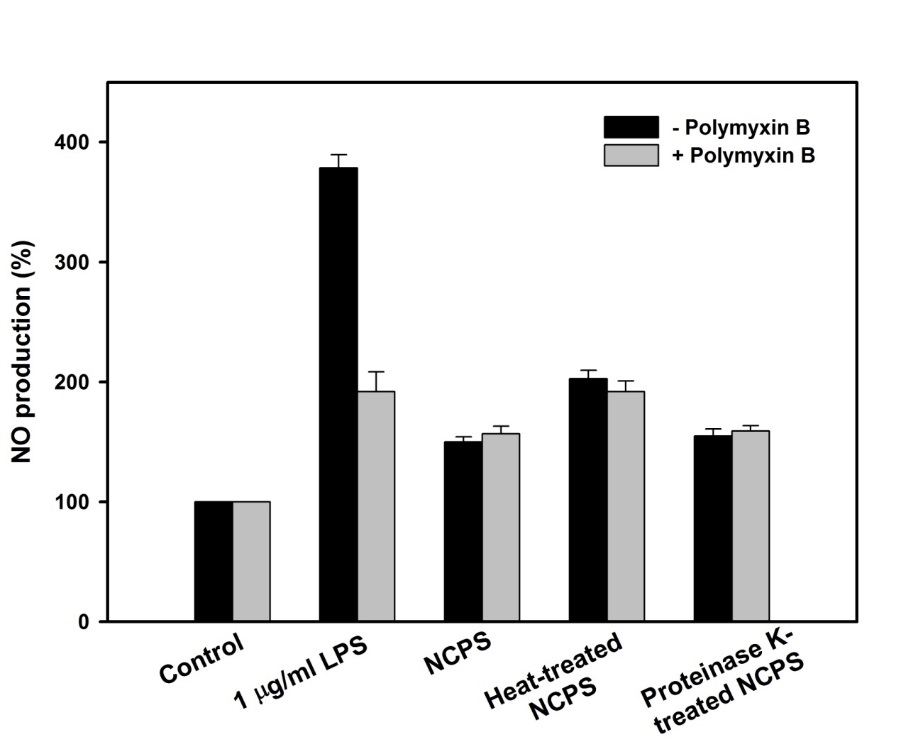


**Figure S3.** Superoxide production of RAW264.7 cells upon the treatments of NCPS and polymyxin B.
